# Sequence diversification in recessive alleles of two host factor genes suggests adaptive selection for bymovirus resistance in cultivated barley from East Asia

**DOI:** 10.1007/s00122-016-2814-z

**Published:** 2016-11-09

**Authors:** Ping Yang, Antje Habekuß, Bernhard J. Hofinger, Kostya Kanyuka, Benjamin Kilian, Andreas Graner, Frank Ordon, Nils Stein

**Affiliations:** 1 Leibniz Institute of Plant Genetics and Crop Plant Research (IPK), OT Gatersleben, 06466 Stadt Seeland, Germany; 2 Institute for Resistance Research and Stress Tolerance, Julius Kuehn Institute (JKI), Federal Research Centre for Cultivated Plants, 06484 Quedlinburg, Germany; 3 Plant Biology and Crop Science Department, Rothamsted Research, Harpenden, AL5 2JQ UK; 4 Global Crop Diversity Trust, Platz der Vereinten Nationen 7, 53113 Bonn, Germany

## Abstract

**Key message:**

**Two distinct patterns of sequence diversity for the recessive alleles of two host factors** ***HvPDIL5*** **-** ***1*** **and** ***HvEIF4E*** **indicated the adaptive selection for bymovirus resistance in cultivated barley from East Asia.**

**Abstract:**

Plant pathogens are constantly challenging plant fitness and driving resistance gene evolution in host species. Little is known about the evolution of sequence diversity in host recessive resistance genes that interact with plant viruses. Here, by combining previously published and newly generated targeted re-sequencing information, we systematically analyzed natural variation in a broad collection of wild (*Hordeum spontaneum*; *Hs*) and domesticated barleys (*Hordeum vulgare*; *Hv*) using the full-length coding sequence of the two host factor genes, *HvPDIL5*-*1* and *HvEIF4E*, conferring recessive resistance to the agriculturally important *Barley yellow mosaic virus* (BaYMV) and *Barley mild mosaic virus* (BaMMV). Interestingly, two types of gene evolution conferred by sequence variation in domesticated barley, but not in wild barley were observed. Whereas resistance-conferring alleles of *HvEIF4E* exclusively contained non-synonymous amino acid substitutions (including in-frame sequence deletions and insertions), loss-of-function alleles were predominantly responsible for the *HvPDIL5*-*1* conferred bymovirus resistance. A strong correlation between the geographic origin and the frequency of barley accessions carrying resistance-conferring alleles was evident for each of the two host factor genes, indicating adaptive selection for bymovirus resistance in cultivated barley from East Asia.

**Electronic supplementary material:**

The online version of this article (doi:10.1007/s00122-016-2814-z) contains supplementary material, which is available to authorized users.

## Introduction

Plant pathogens such as viruses, bacteria and fungi are constantly challenging crop plant fitness since infections may cause yield losses (Oerke 2006). Resistant genotypes have the selective advantage of producing (more) seeds, thus their offspring may predominate in populations after some generations under strong selection. Some pathogen isolates may counteract this via own rapid evolution through mutation—this triggers the ‘arms race’ or the antagonistic co-evolution between plants and their adapted pathogens (Jones and Dangl 2006; Obbard and Dudas 2014; Rausher 2001). In natural populations or wild species a higher level of genetic diversity could maintain natural resistances that prevent the epidemic outbreak of diseases and their severe consequences for the populations or species (Tanksley and McCouch 1997). By contrast, the monoculture in agricultural systems destabilizes the balance, and the densely grown and genetically uniform crop populations facilitate the success of individual newly evolved and virulent strains of a pathogen supporting the chance of pathogen transmission and epidemic outbreak of respective diseases (Stukenbrock and McDonald 2008). Therefore, understanding the patterns of sequence variation underlying the evolution of dominant resistance genes and recessive host factors in cultivated crops and their wild progenitors should contribute to reveal the evolutionary processes during domestication, improvement and local adaptation of crops and thus may provide insight into a more sustainable use of resistance resources in agriculture.

Unlike bacterial and fungal pathogens that maintain relatively large genomes, plant RNA viruses have small genomes and commonly encode only for about four to ten viral proteins. Such viruses cannot complete their entire proliferation cycle independently of their plant hosts. For instance, they are dependent on the host’s cellular protein synthesis machinery and also often employ other host-encoded proteins to complete their lifecycle (Ahlquist et al. 2003; Robaglia and Caranta 2006). These host components are known as ‘susceptibility’ or ‘host’ factors. Mutations in the genes coding for host factors may result in virus resistance (Soosaar et al. 2005; Whitham and Wang 2004). These recessive resistance genes, therefore, function very differently compared to the dominant resistance genes such as the nucleotide binding leucine rich repeat (NB-LRR) protein encoded genes (Sacristan and Garcia-Arenal 2008), which recognize directly or indirectly the so-called effector proteins produced by the pathogens and then trigger an active defense response often culminating in plant cell death and halting the pathogen growth (Tiffin and Moeller 2006). Depending on the selection pressure imposed by the frequency and/or severity of the respective virus disease, sequence variation in the host genes conferring disease resistance may be quickly selected and maintained at higher frequency in populations under conditions of natural selection. Artificial selection during domestication, adaptive improvement and breeding could even accelerate this selection process. Rapid evolution of plant virus genomes, which occurs constantly at least in RNA viruses due to the lack of an efficient RNA sequence repair system (Domingo and Holland 1997), may result in emergence of new virus pathotypes being able to overcome the newly evolved plant defense mechanism, e.g. by cooperating with alternate host factors or by adapting to the modified host proteins (Kühne et al. 2003; Okada et al. 2003). In agricultural systems, infectious diseases reduce yields and can threaten the sustainability of crop production (Oerke 2006). Selection for resistance forms of host factors was probably unintentional during the early domestication and an adaptation to local environments (Kang et al. 2005). Understanding the natural variation of such host factors could reveal the mechanisms underlying the ‘arms race’ between cultivated plants and their viruses.

The barley yellow mosaic virus disease, wherever it occurs, is a severe, long-term and persistent biotic stress, threatening winter barley production in a majority of European and Eastern Asian countries (Kühne 2009). It was first detected in Japan in the 1940s (Ikata and Kawai 1940), and later in the 1950s observed in China and Korea (Lee et al. 1996; Zhou and Cao 1985), and since 1978 reported to occur in European countries (Kühne 2009). The disease is caused by infection with isolates of *Barley yellow mosaic virus* and/or *Barley mild mosaic virus*, two members of the genus *Bymovirus* in the *Potyviridae* family. Under natural conditions, the bymovirus disease is transmitted through barley roots by the soil-borne Plasmodiophorid *Polymyxa graminis* (Adams et al. 1988). Persistent resting spores of this microorganism provide protection for virus particles, which can remain infectious for many years (Huth 1991). There are currently no chemicals available for control of plant viruses, whereas use of chemical agents (i.e. soil fumigants) effective against the virus vector, *P. graminis*, is undesirable due to ecological implications (Kanyuka et al. 2003; Kühne 2009). After infection, yield losses of up to 50% have been observed in Europe (Plumb et al. 1986). In Asia, the disease can be even more severe where complete yield losses were recorded in China in the mid-1970s (Chen 2005; Kühne 2009). Breeding for genetic resistance to bymoviruses, therefore, remains the most viable and environmentally safe option for crop protection, and over time eighteen bymovirus resistance loci were genetically characterized in barley (Kai et al. 2012; Ordon et al. 2005). We previously isolated by positional cloning two recessive resistance genes *rym1/11* and *rym4/5*, corresponding to two different host factor genes *Protein Disulfide Isomerase Like 5*-*1* (*HvPDIL5*-*1*) and *Eukaryotic Translation Initiation Factor 4E* (*HvEIF4E*), respectively (Kanyuka et al. 2005; Stein et al. 2005; Yang et al. 2014a, b). The resistance genes/alleles *rym1* and *rym5* were originally found in a Chinese landrace “Mokusseko 3” and shown to be inherited independently (Konishi et al. 1997). The HvEIF4E protein putatively functions in assisting the translation initiation of bymovirus precursor protein (Kanyuka et al. 2005; Stein et al. 2005), and HvPDIL5-1 is speculated to function as chaperone in correct folding of virus proteins (Yang et al. 2014b). Our previous work revealed sequence diversity of *HvEIF4E* in domesticated barley accessions and *HvPDIL5*-*1* in wild/domesticated accessions (Hofinger et al. 2011; Yang et al. 2014b). However, the sequence diversity of *HvEIF4E* in wild barley was unknown, and importantly, the responses of most of these *HvEIF4E* and *HvPDIL5*-*1* haplotypes to the bymovirus disease of barley were not yet investigated. This limited our understanding of the relevance of naturally occurring sequence variation of both host factor genes in eco-system vs agricultural system, especially in the regions severely threatened by the bymovirus disease. By combining previously published and newly generated sequences and by testing with a viral assay the identified haplotypes, here we systematically investigated the diversity and frequency of sequence variation patterns in resistance-conferring haplotypes of these two host factor genes in barley genotypes from different geographic regions.

## Materials and methods

### Plant materials

365 wild and 2557 domesticated barleys (mostly landraces or historic cultivars, but also including six-rowed barley with brittle rachis *Hordeum vulgare* subsp. *agriocrithon*) with the GPS-coordinated geographical information, which were mainly collected by IPK-Genebank or obtained from the National Small Grains Collection, USDA-ARS (Aberdeen, Idaho, USA), were used in the present study (Supplementary Fig. S1; Supplementary Tables S1, S2). The wild barley accessions were mainly collected from the Near East, and the cultivated barley accessions originated from 89 countries representing most of the barley cultivation areas worldwide. For a small number of accessions the precise collection site was unclear, and for these we specified the latitude and longitude of the capital of the source state/province. Details of the barley accessions and the collection sites (Country, latitude and longitude) are given in Supplementary Table S2.

### Amplicon sequencing

Plant DNA and RNA extraction, synthesis of first-strand cDNA, PCR amplification, purification of PCR products and Sanger sequencing were performed as described previously (Yang et al. 2014b). Purification of PCR amplicons was carried out using the NucleoFast 96 PCR Kit (Macherey-Nagel, Germany). Two µl of each purified PCR product was analyzed and quantified by agarose gel electrophoresis. The optimal amount of each purified amplicon (10 ng per each 100 bp) was subjected to cycle-sequencing and the products analyzed on an ABI-3730xl DNA Analyzer (Applied Biosystems, Darmstadt, Germany). The trimmed sequence reads were assembled using Sequencher 4.7 (Gene Codes, USA). The primers used in this study are given in Supplementary Table S3.

### Resistance tests

Plant material required for performing virus resistance tests was cultivated in climatic chamber as previously described (Habekuß et al. 2008; Yang et al. 2014b). A subset of wild and domesticated barley accessions carrying non-synonymous mutations in either *HvPDIL5*-*1* or *HvEIF4E* was analyzed after mechanical inoculation with the Aschersleben isolate of BaMMV (BaMMV-ASL). At least 10 plants per accession were tested. At three-leaf stage (2 week after sowing) plants were mechanically inoculated twice at an interval of 5–7 days. Four to six weeks later, the phenotypes were collected by detecting viral RNAs by RT-PCR (via amplification of a 525-bp viral VPg gene fragment), or DAS-ELISA with a viral coat protein-specific antibody (Clark and Adams 1977). For instance, if absorbance at 405 nm was below 0.1, the respective plant was scored as being resistant (no virus particles detected). Otherwise, plants were scored susceptible (absorbance >0.1).

### Data analysis

Sequence assembly and alignments were performed using Sequencher v4.7 (Gene Codes, USA). Sequence reads that originated from introns, and those of poor quality or indication of heterozygosity were removed manually from the analysis. The genotypes for which only the partial gene coding sequences were obtained were also excluded from the analysis. The polymorphic loci including loss-of-function (*LoF*), synonymous (*S*) and non-synonymous (*Ns*) were counted manually. The allelic haplotypes, the haplotype diversity (*H*), the nucleotide diversity (*π*) as well as statistics for selection (Fu and Li’s D* and F*, Tajima’s D) were calculated using DNASP v5.10.01 (Librado and Rozas 2009). The generated files, containing polymorphism and haplotype information, were subjected to the Median-Joining network analysis using DNA Alignment v1.3.1.1, Network v4.6.1.1 and Network Publisher v1.3.0.0 software (Fluxus Technology, UK). The significance of type of mutations between wild and domesticated barleys was analysed using Fisher’s Exact Test.

The topographic maps were drawn using ArcGIS 10 software (ESRI, Redlands, CA, USA). The Base Map at 10-km resolution developed by the US Geological Survey (USGS) according to the GTOPO30 global digital elevation dataset (http://eros.usgs.gov) was used. These accessions used in this study were assigned to the Base Map according to the specified collection sites (Supplementary Table S2).

## Results

### *HvPDIL5*-*1* and *HvEIF4E* exhibit distinct patterns of sequence diversity in domesticated but not in wild barley

**Fig. 1:**
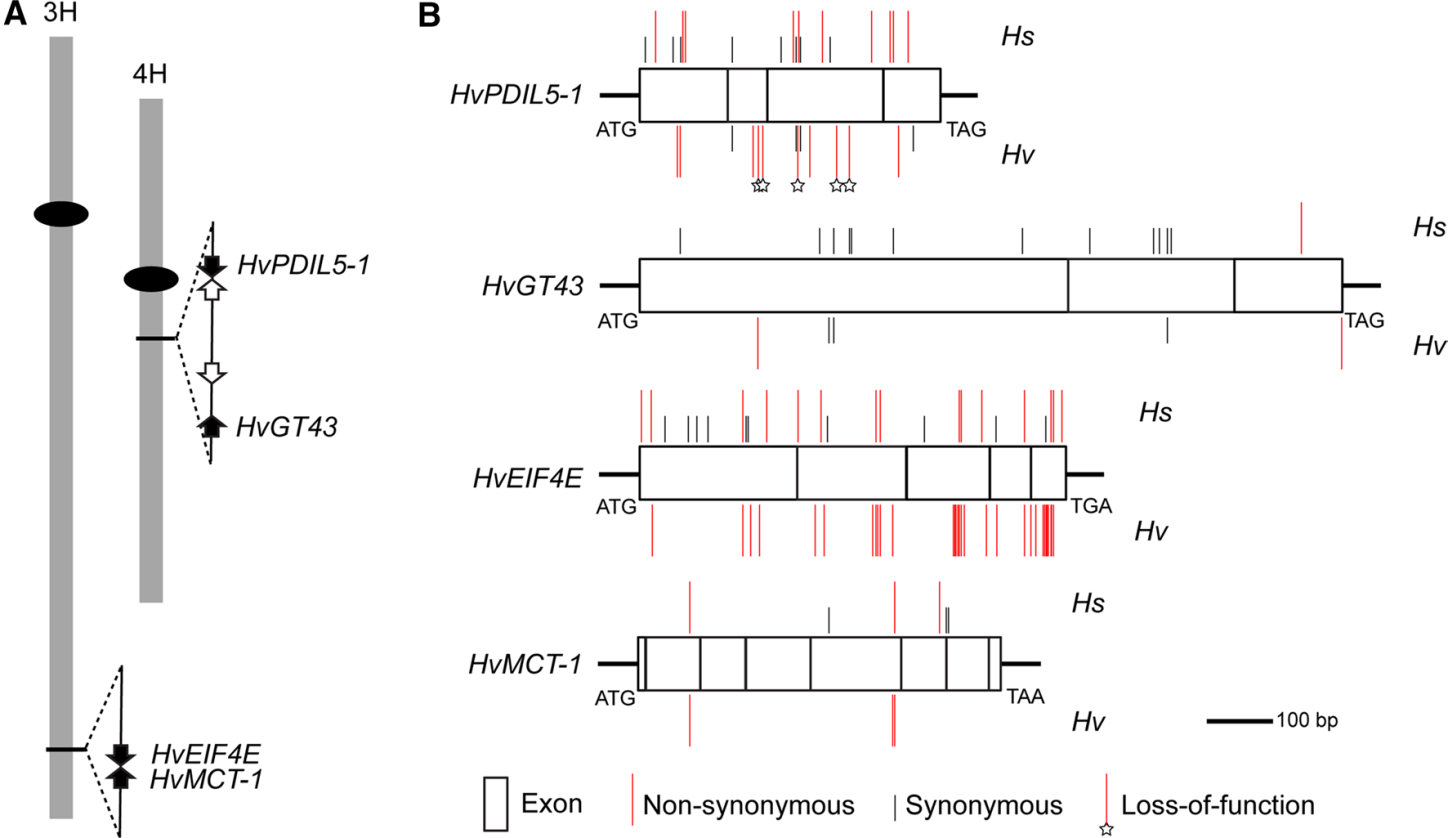
Sequence variation in *HvPDIL5*-*1*, *HvGT43*, *HvEIF4E* and *HvMCT*-*1* in wild (*Hordeum spontaneum*; *Hs*) and domesticated (*Hordeum vulgare*; *Hv*) barley. **a** Physical positions of these four genes on the barley chromosomes 3H and 4H. The *solid black ovals* represent centromeres. The distance between *HvEIF4E* and *HvMCT*-*1* is 1048-bp, while the space between *HvPDIL5*-*1* and *HvGT43* is about 620 kb. However, no recombination events were detected in pairs of *HvPDIL5*-*1*/*HvGT43* and *HvEIF4E*/*HvMCT*-*1* in thousands of segregating F_2_ lines (Stein et al. 2005; Yang et al. 2014a, b). **b** Distribution of mutations in the full-length open reading frame (fl-ORFs). The diagrams were drawn according to the length of each fl-ORF. The *rectangles* within each fl-ORF show positions of gene coding exons

To assess the extent and pattern of global genetic diversity of the two host factor genes *HvPDIL5*-*1* and *HvEIF4E*, sequence variation in the full-length open reading frames (fl-ORFs) of both genes was surveyed in 365 wild and 2557 domesticated geographically referenced barley accessions (Supplementary Tables S1, S2). The dataset included (i) previously published sequences of *HvEIF4E* obtained from 1090 cultivated barleys (Hofinger et al. 2011) and *HvPDIL5*-*1* obtained from 350 and 1382 wild and domesticated barleys, respectively (Yang et al. 2014b), as well as (ii) newly generated sequences of *HvPDIL5*-*1* and *HvEIF4E* obtained in this study from 856 cultivated and 320 wild barley accessions, respectively (Table 1). Furthermore, we sequenced in a subset of the total of 2922 barley accessions the fl-ORFs of *HvGT43* and *HvMCT*-*1* (Supplementary Table S1). These genes co-segregated and are located in close physical proximity to *HvPDIL5*-*1* and *HvEIF4E*, respectively (Fig. 1a, Stein et al. 2005; Yang et al. 2014a, b), and were included in this study for the purpose of determining the demography of population and testing the effect of genomic location on patterns of sequence diversity. The relative genomic positions of *HvGT43/HvPDIL5*-*1* and *HvMCT*-*1*/*HvEIF4E*, besides allocating to different chromosomes, are significantly different. The first gene pair is located close to the centromere (low recombination frequency region) on the long arm of chromosome 4H (4HL) whereas the second gene pair is located close to the telomere (high recombination frequency region) on the long arm of chromosome 3H (3HL) (IBRC 2012). Importantly, fl-ORF sequences of *HvPDIL5*-*1* and *HvEIF4E* genes were obtained from a shared set of 1152 genotypes, and fl-ORF sequences of all four genes *HvPDIL5*-*1*, *HvEIF4E*, *HvGT43* and *HvMCT*-*1* were obtained from a shared set of 392 barley accessions (Supplementary Table S2).

**Table 1:** Sequence diversity and selection statistics of four genes *PDIL5*-*1*, *GT43*, *EIF4E* and *MCT*-*1* in wild and domesticated barley

Genes	Length of fl-ORFs	No. of fl-ORFs	Polymorphisms			No. of haplotypes	*H*	*π*	D*	F*	*Tajima’s D*	Origin of sequences
			*LoF*	*Ns*	*S*							
*PDIL5*-*1_Hs*	456	350	0	10	8	19	0.26430	0.00077	−1.88648	−2.42180*	−2.17856**	(1)
*PDIL5*-*1_Hv*		2238	5	5	4	15	0.05300	0.00013	−2.66316*	−2.87123*	−1.78450	This study + (1)
*GT43_Hs*	1065	192	0	1	12	14	0.56200	0.00069	−1.82707	−2.11640	−1.66355	This study
*GT43_Hv*		221	0	2	3	7	0.63200	0.00080	−1.73015	−1.18574	0.46450	This study
*eIF4E_Hs*	648	320	0	14	11	26	0.57420	0.00140	−1.69810	−2.25851	−2.10362*	This study
*eIF4E_Hv*		1090	0	30	0	47	0.61000	0.00216	−3.23898*	−3.16410**	−1.81117*	(2)
*MCT*-*1_Hs*	546	182	0	3	3	7	0.21300	0.00040	−3.03527*	−3.03194*	−1.61976	This study
*MCT*-*1_Hv*		271	0	3	0	4	0.09900	0.00018	−2.33294*	−2.33602*	−1.21970	This study

(1) and (2), retrieved from Yang et al. (2014b) and Hofinger et al. (2011), respectively. Loss-of-function variation (*LoF*), non-synonymous (*Ns*), synonymous (*S*), haplotype diversity (*H*), nucleotide diversity (*π*), Fu and Li’s *D*-test statistics (D*), Fu and Li’s *F*-test statistics (F*), Tajima’s *D*. The parameters (*H*, *π*, D*, F*, Tajima’s *D*) are calculated using DnaSP v5.10.01 with the gaps not-considered algorithm

* *P* ≤ 0.05; ** *P* ≤ 0.02

**Fig. 2:**
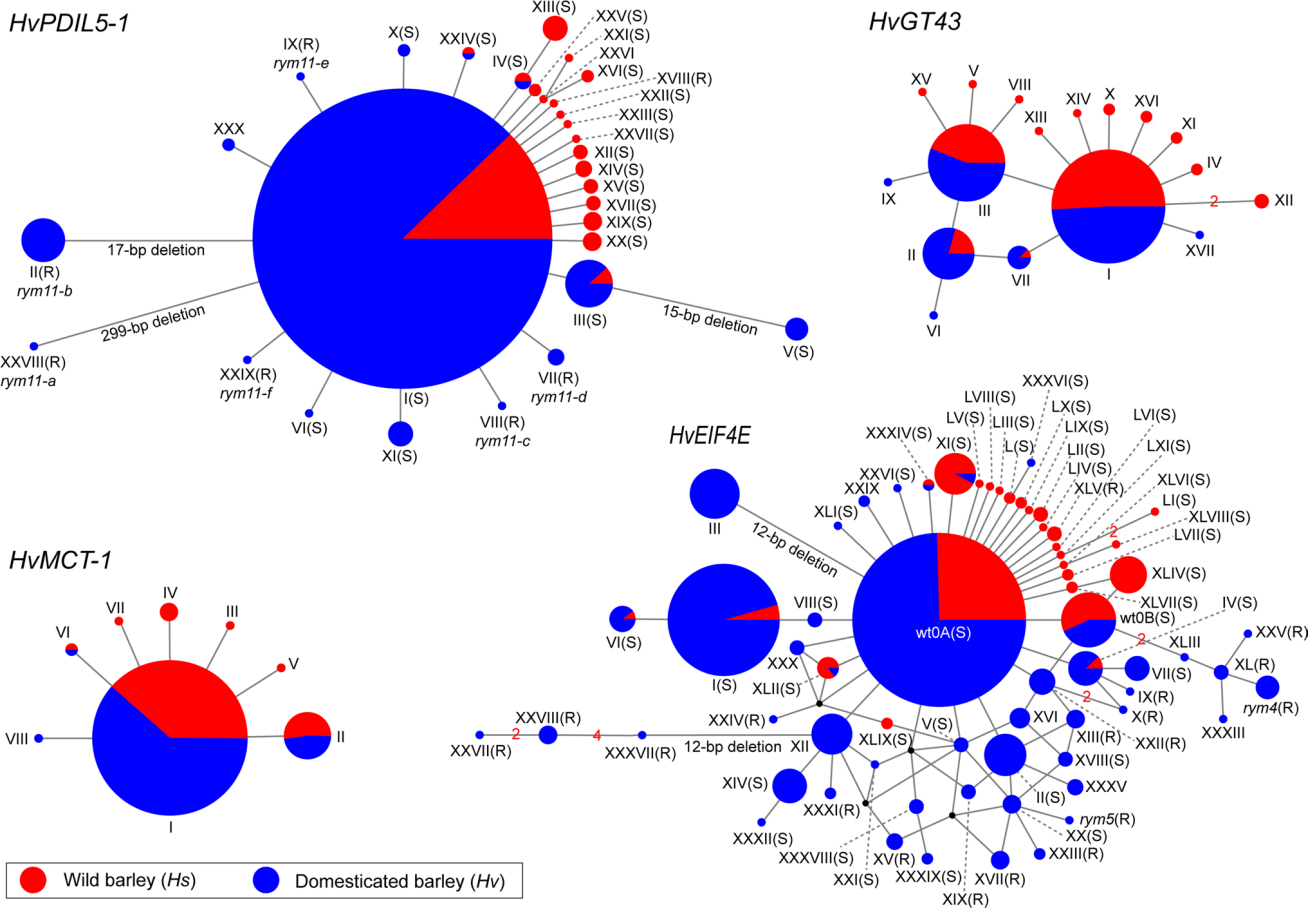
Haplotype networks of *HvPDIL5*-*1*, *HvGT43*, *HvEIF4E* and *HvMCT*-*1*. Median-Joining (MJ) network was constructed from the haplotypes that were defined by the full-length ORFs. Haplotypes are represented by *blue*, *red* and *blue*/*red circles*. Size of each *circle* corresponds to the number of the accessions identified to carry that particular haplotype. *Short solid lines* represent genetic distances between haplotypes, typically indicating in a single base pair difference. More substantial differences are represented by longer *solid lines* with the nature of polymorphism indicated below each *line*. *(R)* and *(S)* indicate the bymovirus resistance and susceptibility conferring haplotypes, respectively. For further details of each haplotype please see Supplementary Tables S4–S8 (color figure online)

After removal of low quality data a total of 2588, 1410, 413 and 453 fl-ORF sequences of the four genes *HvPDIL5*-*1*, *HvEIF4E*, *HvGT43* and *HvMCT*-*1* were collected and analyzed for sequence diversity, respectively (Fig. 1; Table 1). A total of 30, 65, 17 and 8 haplotypes, respectively, were defined for *HvPDIL5*-*1*, *HvEIF4E*, *HvGT43* and *HvMCT*-*1* (Fig. 2; Supplementary Tables S4–S8). This included two newly identified *HvPDIL5*-*1* haplotypes (hap-XXIX and XXX) and 18 newly identified *HvEIF4E* haplotypes (hap-XLIV–LXI) (Supplementary Tables S4, S5). One predominating haplotype each was found for *HvPDIL5*-*1* (hap-I, 94.5%) and for *HvMCT*-*1* (hap-I, 92.3%), respectively, whereas two and three frequent haplotypes were detected for *HvEIF4E* (hap-wt0A, 57.1%; hap-I, 17.6%) and *HvGT43* (hap-I, 55.9%; hap-II, 11.6%; hap-III, 25.9%) (Supplementary Tables S4–S8).

We further analyzed the coding sequence diversity of these four genes in wild barley in comparison to domesticated barley.

#### HvPDIL5-1

Nineteen wild barley haplotypes (350 analyzed sequences) were observed based on ten non-synonymous (refers to non-synonymous amino acid change or in-frame deletions/insertions) and eight synonymous mutations, while only 15 haplotypes in domesticated barley (from 2238 sequences) were revealed with five containing loss-of-function mutations/deletions, another five containing non-synonymous mutations and remaining four containing synonymous mutations (Table 1). Synonymous and non-synonymous mutations in wild barley occurred evenly along the coding gene sequence, whereas loss-of-function sequence variation in domesticated barley were enriched in the central part of the gene (Fig. 1b), coding putatively for the functional thioredoxin domain (Yang et al. 2014b). Four haplotypes (hap-I, III, IV and XXIV) were shared between wild and domesticated barleys (Fig. 2). Haplotype I predominated in the collection and other haplotypes were derived from this ancestral haplotype by one or two mutations (Yang et al. 2014b). Interestingly, loss-of-function haplotypes were only found to be significantly accumulated in domesticated, but not in wild barley (Fisher’s Exact Test, *P* = 0.0099). The number of polymorphisms, haplotypes, as well as the values of the parameters *H* and *π* were lower in domesticated barley compared to those in wild barley (Table 1), indicating the reduction of gene sequence diversity. Statistical tests employing Fu and Li’s D* and F* and also Tajima`s *D* indicated low frequencies of rare alleles of *HvPDIL5*-*1* in wild and domesticated barley, possibly being related to selection or population size expansion.

#### HvGT43

The close neighbor gene of *HvPDIL5*-*1* exhibited thirteen and five polymorphic nucleotide positions (including non-synonymous and synonymous mutations) in 192 wild and 221 domesticated barley accessions, respectively, defining 14 and seven haplotypes (Table 1). Since the same three major haplotypes (hap-I, II and III) were also present in domesticated barley accessions (95.5%, 211 out of 221 accessions), no strong differences were found for *H* and *π* values between the domesticated and wild barleys (Table 1). The same set of cultivated barley *vs* wild barley revealed a strongly decreased sequence diversity at the *HvPDIL5*-*1* locus (Supplementary Table S9). This indicated that the observed sequence diversity of *HvPDIL5*-*1* and *HvGT43* was gene specific and irrespective of chromosomal position. Importantly, no defective haplotype was found at the *HvGT43* locus.

#### HvEIF4E

In 320 wild and 1090 domesticated barley accessions, 26 and 47 haplotypes were defined, respectively. Fourteen non-synonymous and eleven synonymous nucleotide polymorphisms were detected in wild barley (Table 1), and these were evenly distributed along the coding gene sequence (Fig. 1b). This pattern was similar to the observations at the *HvPDIL5*-*1* locus, which implied a lack of specific selection at a particular region of both genes in wild barley. As to domesticated barley, an extreme situation was observed: sequence diversity was caused by a total of 30 non-synonymous mutations, whereas not even a single silent (synonymous) mutation was detected (Fisher’s Exact Test, *P* = 0.0001, Table 1). Non-synonymous mutations were enriched at three regions (nucleotides 336–384, 480–528, and 600–648) (Fig. 1b), coding for amino acid sequences located in proximity to the putative cap-binding domain of the protein (Kanyuka et al. 2005; Stein et al. 2005). In contrast to *HvPDIL5*-*1* that showed a simple evolutionary network, the Median-Joining (MJ) network revealed a complex status of sequence variation for *HvEIF4E* (Fig. 2). For instance, it seems likely that haplotype XII originated by one additional mutation from one of haplotypes wt0A, XIV, XXI, XXXI or XXXII. The number of polymorphic loci and haplotypes, and *H* and *π* values in cultivated barley were not lower than in wild barley.

#### HvMCT-1

Three synonymous and three non-synonymous mutations of this gene were detected in wild barley accessions, while only three non-synonymous mutations were found in domesticated barley (Table 1). In contrast to *HvEIF4E* that represented a higher sequence diversity in domesticated barley, the same set of barley accessions revealed dramatically decreased sequence diversity at the *MCT*-*1* locus in domesticated *vs* wild barley (Supplementary Table S9). This indicated that the higher sequence diversity of *HvEIF4E* was not a function of recombination frequency at the 3HL telomeric region.

Collectively, the sequence variation analyses revealed two qualitatively distinct patterns of gene sequence diversity with a bias for loss-of-function mutations in *HvPDIL5*-*1* and non-synonymous mutations in *HvEIF4E—*both occurring only in domesticated but not in wild barley accessions. Importantly, analyzing the sequence diversity of the next neighboring genes *HvGT43* and *HvMCT*-*1* indicated that the two distinct patterns observed for *HvPDIL5*-*1* and *HvEIF4E* are characteristic for the respective positions on the chromosome.

Bymovirus resistance correlated with the increased diversity of the host factor genes *HvEIF4E* and *HvPDIL5*-*1.*

The barley yellow mosaic virus disease is a highly virulent and persistent biotic stress for cultivated barley in East Asia and Europe (Kühne 2009), and selection for resistance was an important target in recent barley breeding and cultivation in these regions (Chen 2005; Ordon et al. 2005). To determine whether appearance of haplotypes conferring virus resistance was associated with the occurrence of loss-of-function and non-synonymous exchange mutations in *HvPDIL5*-*1* and *HvEIF4E* (the corresponding recessive alleles of these genes conferring resistance to bymoviruses are known as *rym1/11* and *rym4/5*, respectively), we tested accessions carrying different haplotypes at the *HvPDIL5*-*1* or *HvEIF4E* locus for their reaction to a common isolate of *Barley mild mosaic virus* (BaMMV-ASL). A total of 30 and 65 different haplotypes of *HvPDIL5*-*1* and *HvEIF4E*, respectively, were defined in wild and domesticated barley accessions (Supplementary Tables S4, S5). Of these, a subset of accessions representative for haplotypes carrying either loss-of-function and/or non-synonymous mutations were subjected to mechanical virus inoculation (Supplementary Tables S10, S11). In these assays, primarily the accessions carrying known, previously characterized susceptibility haplotypes at one locus and novel haplotypes with the unknown susceptibility/resistance status at another locus were used. For instance, when one accession contained a known susceptibility haplotype of *HvPDIL5*-*1*, testing the susceptibility/resistance status of a novel haplotype of *HvEIF4E* was allowed. However, most of accessions carrying haplotypes of unknown susceptibility/resistance status at both loci (*HvPDIL5*-*1* and *HvEIF4E*) were excluded (or the phenotype of respective haplotype was marked as ‘unknown’). It needs to be noted, that this approach cannot rule out completely the remote possibility that an accession may contain additional independent bymovirus resistance loci. Less than 1% of nearly 10,000 domesticated barley accessions were reported to be completely resistant against isolates of BaYMV and BaMMV in the field in earlier studies (Ruan et al. 1984; Zhou and Cao 1985).

#### HvPDIL5-1

**Fig. 3:**
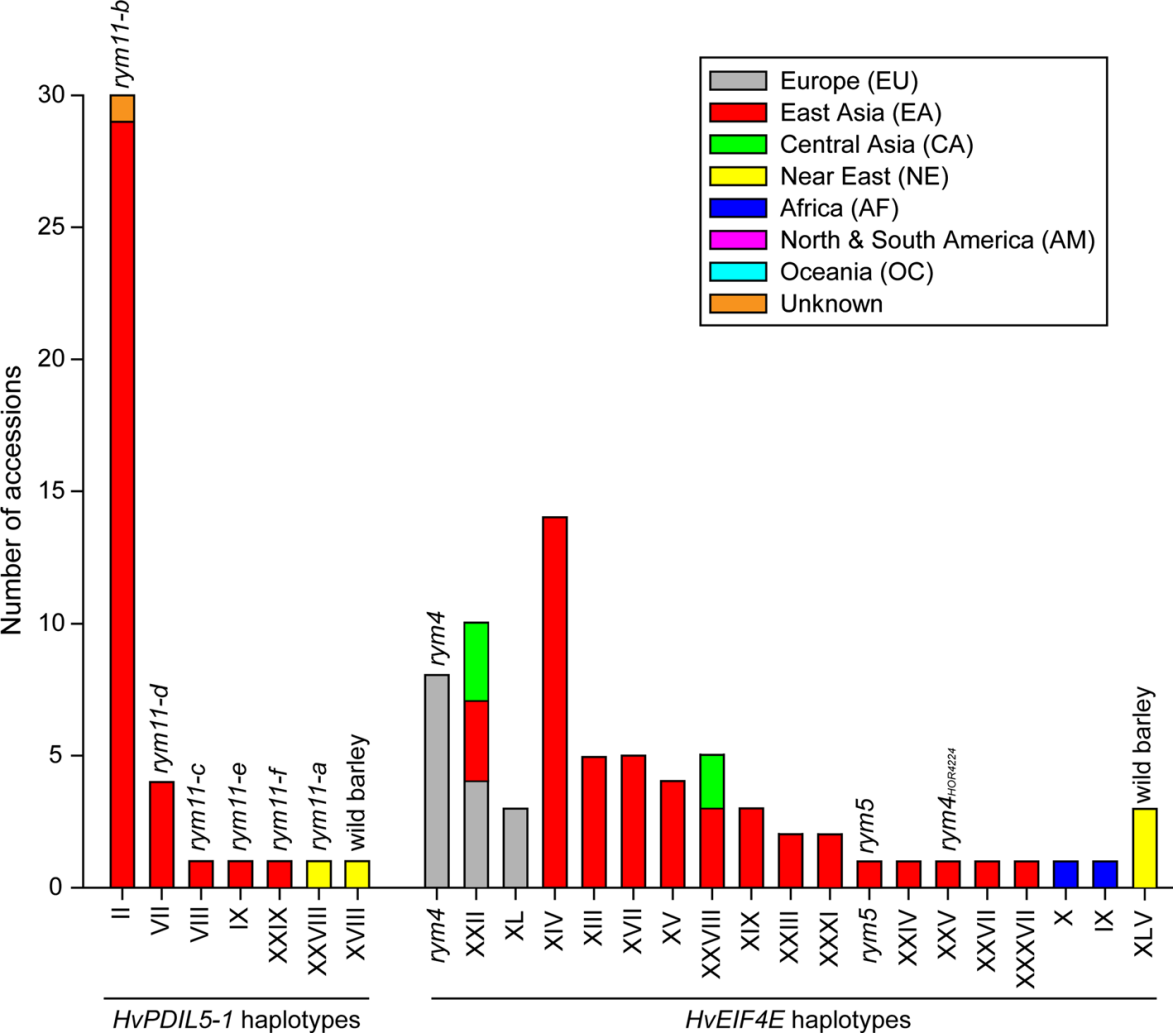
Geographic distribution of barley accessions carrying resistance haplotypes of *HvPDIL5*-*1* and *HvEIF4E* against BaMMV-ASL. *Bars* indicate the total number of accessions carrying the same resistance-conferring haplotype

**Fig. 4:**
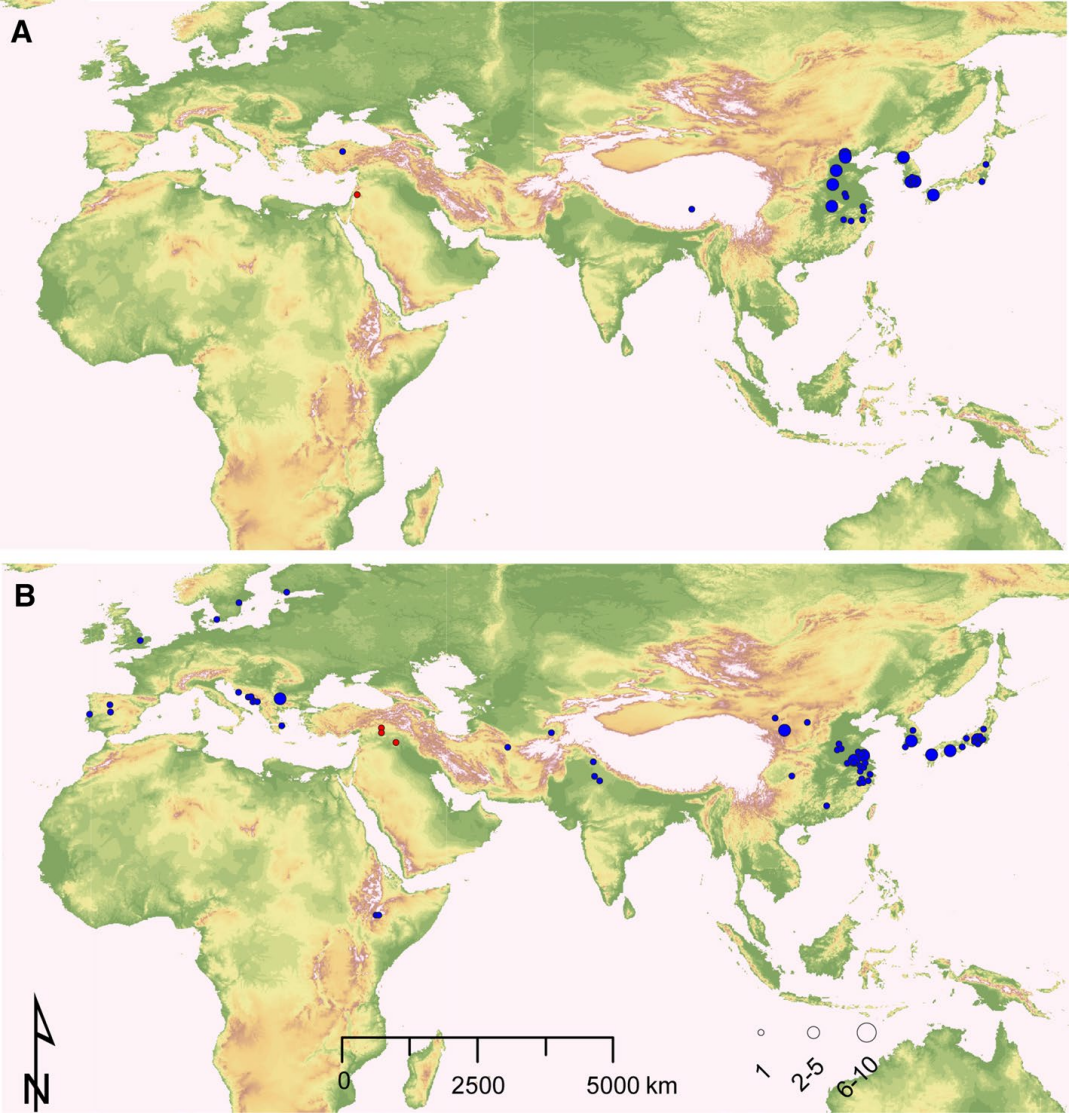
Collection sites of the barley accessions carrying resistance-conferring haplotypes of *HvPDIL5*-*1* (**a**) and *HvEIF4E* (**b**). Each accession is allocated according to latitude and longitude coordinates of its geographic collection site (represented as *red* or *blue circles*). *Red* and *blue circles* indicate collection sites for wild and domesticated barleys, respectively. Size of each *circle* represents the number of accessions collected at each particular collection site (color figure online)

Seven *HvPDIL5*-*1* haplotypes (hap-II, VII, VIII, IX, XVIII, XXVIII, and XXIX) were classified as resistant to BaMMV-ASL (Fig. 3; Supplementary Table S4). This included a previously undescribed loss-of-function haplotype (G256A/hap-XXIX/*rym11*-*f*) encoding a truncated PDIL5-1-like protein, and a known haplotype (hap-XVIII) containing a non-synonymous mutation (A239G/Glu80Gly) in *HvPDIL5*-*1*. Only one wild barley accession carrying hap-XVIII was identified, and it showed resistance to BaMMV-ASL (Supplementary Table S10). This reveals *H. spontaneum* as a useful source of resistance to bymoviruses. In domesticated barley, accessions carrying one of the six *HvPDIL5*-*1* haplotypes, hap-II, VII, VIII, IX, XXVIII, and XXIX, also displayed resistance to BaMMV-ASL (Fig. 3). These resistance-conferring haplotypes were present in 38 (1.69%) of 2238 cultivated barley accessions (Figs. 3, 4a), and of these 37 were carrying haplotypes containing loss-of-function mutations in *HvPDIL5*-*1* (Supplementary Table S4). Thus, in the case of *HvPDIL5*-*1*, it appears that the mutations resulting in a loss of function of the encoded host factor protein are largely responsible for the observed bymovirus resistance phenotype.

#### HvEIF4E

Accessions representing either one of the identified nineteen *HvEIF4E* haplotypes were tested as conferring resistance to BaMMV-ASL (Supplementary Tables S5, S11). This included three previously reported resistance alleles (*rym4*, *rym5*, and *rym4*_*HOR4224*_) (Perovic et al. 2014; Stein et al. 2005), and 15 new haplotypes for which bymovirus resistance had not been reported before (hap-IX, X, XIII, XIV, XV, XVII, XIX, XXIII, XXIII, XXIV, XXVII, XXVIII, XXXI, XXXVII, XLI, and XLV) (Fig. 3). Wild barley accessions carrying *HvEIF4E* hap-XLV were also resistant to BaMMV-ASL (Supplementary Table S11), confirming that *H. spontaneum* is a useful source of resistance to bymoviruses. In domesticated barley, sixty-eight (5.31%) of 1090 accessions carried any of the remaining 18 resistance-conferring *HvEIF4E* haplotypes (Fig. 3). These haplotypes were found in many barley cultivation areas except the Americas, Near East and Oceania (Fig. 4b), where no reports about the occurrence of the bymovirus disease are available. Interestingly, all resistance-conferring haplotypes contained non-synonymous mutations in the coding sequence of *HvEIF4E*.

Collectively, the data presented above revealed a strong correlation between bymovirus resistance and either the loss-of-function mutations in *HvPDIL5*-*1* or the non-synonymous mutations in *HvEIF4E*. Importantly, resistance-conferring haplotypes were found preferentially in cultivated barley rather than wild barley, suggesting the more frequent rise of bymovirus resistance after domestication.

Type and frequency of mutations in the two host factor genes in context of geographical origins of the domesticated barley accessions.

The large collection of geographically referenced domesticated barley accessions allowed us to investigate if any geographic region contributed disproportionally to gene sequence diversity (loss-of-function mutations of *HvPDIL5*-*1* and non-synonymous mutations of *HvEIF4E*), and if there were any preferred geographic origins of resistance-conferring haplotypes. Seven sub-populations (Africa, Americas, Central Asia, East Asia, Europe, Near East and Oceania) of the domesticated barley accessions were defined (Supplementary Table S1), and analyzed for diversity in the host factor genes *HvPDIL5*-*1* and *HvEIF4E* (Tables 2, 3).

**Table 2:** Statistics of sequence diversity of *HvPDIL5*-*1* in sub-populations of domesticated barley germplasm

Sub-populations	No. of sequences	No. of resistance accessions	Polymorphisms			No. of haplotypes	No. of resistance haplotypes	*H*	*π*	D*	F*	*Tajima’s D*
			*LoF*	*Ns*	*S*							
Africa	240	0	0	0	1	2	0	0.06500	0.00014	0.44585	0.14167	−0.59760
Americas	231	0	0	0	1	2	0	0.01700	0.00004	0.44760	0.03765	−0.88601
Central Asia	177	0	0	2	1	4	0	0.05600	0.00012	−0.70818	−1.12243	−1.45612
East Asia	442	36	4	2	0	7	5	0.03100	0.00007	−3.30399^**^	−3.23293^**^	−1.52225
Near East	367	1	1	2	3	7	1	0.09000	0.00020	−1.48159	−1.80887	−1.56545
Europe	749	0	0	1	2	4	0	0.04700	0.00011	−1.46884	−1.56068	−0.95383
Oceania	16	0	0	0	1	2	0	0.12500	0.00027	−1.45287	−1.56820	−1.16221

**Table 3:** Statistics of sequence diversity of *HvEIF4E* in sub-populations of domesticated barley germplasm

Sub-populations	No. of sequences	No. of resistance accessions	Polymorphisms			No. of haplotypes	No. of resistance haplotypes	*H*	*π*	D*	F*	*Tajima’s D*
			*LoF*	*Ns*	*S*							
Africa	134	2	0	7	0	8	2	0.59500	0.00190	−1.28382	−1.14694	−0.36743
Americas	204	0	0	9	0	9	0	0.57200	0.00187	−2.56684*	−2.25889	−0.69846
Central Asia	105	5	0	16	0	14	2	0.37400	0.00086	−2.24432	−2.67020*	−2.22980**
East Asia	190	46	0	22	0	27	14	0.79600	0.00269	−2.63509*	−2.59330*	−1.42918
Europe	304	15	0	20	0	22	3	0.50700	0.00180	−1.92887	−2.21970	−1.70488
Near East	139	0	0	10	0	7	0	0.32900	0.00129	−1.60756	−1.80141	−1.33962
Oceania	14	0	0	3	0	3	0	0.52700	0.00166	NA	NA	NA

*NA* not applicable

#### HvPDIL5-1

The polymorphic loci, the number of haplotypes, *H* and *π* of *HvPDIL5*-*1* decreased in all seven sub-populations of domesticated barley compared to those in wild barley (Tables 1, 2). Six *HvPDIL5*-*1* haplotypes (hap-II, VII, VIII, IX, XXVIII and XXIX) were found to be associated with bymovirus resistance, with five of those containing a loss-of-function mutation/deletion and one other carrying a non-synonymous mutation. One of these resistance-conferring haplotypes of *HvPDIL5*-*1*, *rym11*-*a* (hap-XXVIII), carrying a 1375-bp deletion was found only in one accession from Near East (Yang et al. 2014b). The other five haplotypes were found in 36 accessions originated from East Asia and one accession (Russia 57, hap-II, *rym11*-*b*) for which the exact location of a collection site was uncertain (Fig. 3). Of these, 35 contained haplotypes with loss-of-function mutations, indicating a disproportional frequency of resistance-conferring haplotypes in East Asia compared to other barley cultivation regions (Fig. 4a). The frequency of these haplotypes varied between 0.23% (e.g. hap-VIII) and 6.56% (hap-II) in the set of 442 accessions from East Asia used in this study. In comparison, no accessions carrying resistance alleles were found in Europe, where the bymovirus disease is also known to occur (Kühne 2009).

#### HvEIF4E

The genetic diversity of *HvEIF4E* varied among cultivated barley from the different regions of origin (Table 3). The highest diversity was observed in barley from East Asia. Most of the bymovirus resistance-conferring haplotypes were found exclusively in a single barley cultivation area, except hap-XXII and XXVIII, which were present in more than two major geographic regions (Fig. 3). Two haplotypes, *rym4* and *rym5*, were extensively found in Europe and East Asia, respectively (Fig. 3). This is consistent with the known origin of barley landraces Ragusa (from Croatia) and Mokusekko 3 (from China) that were originally identified as carriers of *rym4* and *rym5*, respectively, and used for introgression of bymovirus resistance into elite barley germplasm by breeding (Ordon and Friedt 1993). In East Asia, fourteen resistance-conferring haplotypes were found (Fig. 3). They were present in 46 (24.2%) of 190 cultivated barley accessions from this region in our collection (Table 3). Each haplotype was present at relatively low frequency in the set of accessions tested in this study, ranging between 0.53% (1 of 190, e.g. hap-*rym5*) and 8.95% (14 of 190, hap-XIV). By contrast, in Europe only three haplotypes (hap-*rym4*, XL and XXII) conferring resistance to bymoviruses were found. These haplotypes were present in 15 out of 304 (4.93%) barley accessions from this region (Table 3).

Taken together, the bymovirus resistance-conferring haplotypes caused by loss-of-function mutations in *HvPDIL5*-*1* and non-synonymous mutations in *HvEIF4E* were greatly overrepresented in accessions from East Asia (Tables 2, 3), and the predominant occurrence as minor haplotypes together with statistics indicating selection suggest an evolution of bymovirus resistance alleles in barley germplasm from this geographic region.

## Discussion

This work, by combining previously published data and newly generated sequence information, revealed sequence variation in a broad collection of wild and domesticated barley accessions of the two host factor genes, *HvEIF4E* and *HvPDIL5*-*1*, involved in bymovirus infection. Importantly, by testing haplotypes for resistance, this work demonstrated the two distinct types of sequence diversity patterns occurring in resistance forms of the two host factor genes: non-synonymous mutations in *HvEIF4E* and loss-of-function mutations in *HvPDIL5*-*1*. Re-sequencing of the neighbor genes *HvGT43* and *HvMCT*-*1* in a subset of barley accessions indicated that the two distinct patterns of preferential resistance-conferring sequence polymorphism of the two host factor genes were not caused by population demographic history or their chromosomal positions on the respective chromosomes. Haplotypes associated with bymovirus resistance occurred predominantly in domesticated barley accessions but were rare in wild barley, indicating a rapid rise of resistance in cultivated barley post domestication. Interestingly, sources of bymovirus resistance were mainly found in barley germplasm from East Asia where the barley yellow mosaic virus disease is omnipresent (Kühne 2009), indicating the existence of interaction between bymoviruses and host factors. In addition, although the overall resistance spectrum of the identified *HvEIF4E* and *HvPDIL5*-*1* haplotypes conferring resistance to BaMMV-ASL warrants further investigation (e.g. by testing using a range of BaMMV and BaYMV isolates), these haplotypes represent useful new sources of bymovirus resistance for exploitation in barley breeding programs globally.

The differences in gene diversity patterns observed for *HvEIF4E vs HvPDIL5*-*1* may be explained by the different functions of these two host factor genes.

The eukaryotic translation initiation factor 4E (EIF4E) was repeatedly reported as a host factor involved mainly in potyvirus infections in different plant species (Charron et al. 2008; Wang and Krishnaswamy 2012). The EIF4E protein is of crucial importance in organisms, as it is a component of the conserved eukaryotic translation initiation complex that interacts with the cap-structure of the host mRNAs and enables initiation of the translation of cellular proteins (Browning 1996; Kawaguchi and Bailey-Serres 2002). In previous EcoTILLING (a method for the discovery of natural nucleotide diversity) studies, no haplotypes carrying loss-of-function mutations in *EIF4E* were found in broad collections of accessions of a number of different crops and related wild plant species (Charron et al. 2008; Hofinger et al.^2009^, ^2011^; Ibiza et al. 2010; Konecna et al. 2014; Nieto et al. 2007; Rubio et al. 2009; Stein et al. 2005; Stracke et al. 2007). In the coding sequence of *HvEIF4E*, only non-synonymous mutations were previously identified in an Ethyl Methane Sulfonate (EMS) induced barley population (Gottwald et al. 2009). Consistent with this, a previous EcoTILLING study using cultivated barley also revealed only non-synonymous and small in-frame insertion/deletion mutations in coding sequence of the *HvEIF4E* gene (Hofinger et al. 2011). Similarly, in this study surveying the variation in *HvEIF4E* also in a wild barley collection detected only synonymous and non-synonymous mutations and no loss-of-function mutations. This cumulative evidence strongly suggested that *HvEIF4E* is indispensable in barley, although a highly conserved paralogous gene, *HvEIF(iso)4E*, exists in barley and may potentially provide some functional compensation upon loss-of-function of *HvEIF4E* (Yang et al. 2013). Since the VPg protein of plant viruses in the *Potyviridae* family plays a role similar to that of the eukaryotic mRNA cap-structure and competes with the plant cellular mRNAs for EIF4E binding (Leonard et al. 2000), the changed compatibility by amino acid changes may block the physical interaction with VPg resulting in disease resistance (Charron et al. 2008). This mechanism reported for potyviruses in an interaction with Arabidopsis may explain the situation in barley, since the amino acid substitutions in either HvEIF4E or the bymovirus VPg protein may influence the efficiency of interaction between these proteins (Habekuß et al. 2008; Kanyuka et al. 2005; Kühne et al. 2003; Stein et al. 2005). Thus, we speculate that amino acid substitutions in *HvEIF4E* represent the optimal way of evolution of this gene, to maintain the function for protein translation initiation in barley while simultaneously conferring resistance to bymoviruses.

In contrast to the complex evolutionary haplotype network of *HvEIF4E* in domesticated barley, the *HvPDIL5*-*1* haplotype variation was rather simple. Only 15 haplotypes were found in 2238 cultivated barley accessions. Five haplotypes encoded either no transcript (hap-XXVIII, 1375-bp deletion including first three protein coding exons leads to complete lack of transcript (Yang et al. 2014b), truncated proteins (hap-VII, VIII and XXIX) or shifted coding frames (hap-II). Thus all five identified bymovirus resistance-conferring haplotypes of *HvPDIL5*-*1* lead to non-functional proteins or no protein at all. *HvPDIL5*-*1* belongs to a protein disulfide isomerase (PDI) gene family (Yang et al. 2014b). The number of the family members varied in eukaryotic species. Arabidopsis, rice and maize contain 22, 19 and 22 PDIs, respectively (Houston et al. 2005). Thirty-five barley cultivars from East Asia contained one of these loss-of-function versions of *HvPDIL5*-*1*, suggesting that the non-functional alleles unlikely resulted in yield penalties. In addition, the EMS-induced non-functional alleles (*rym11*-*9699* and -*10253*) of *HvPDIL5*-*1* were not associated with any visible phenotypic changes in plant growth or development (Yang et al. 2014b), suggesting a certain level of dispensability for *HvPDIL5*-*1* in barley. Although an amino acid substitution (*rym11*-*e*) of *HvPDIL5*-*1* can confer bymovirus resistance in domesticated barley, the resistance spectrum of this allele to multiple isolates of bymoviruses still remains to be determined (Yang et al. 2014a). Overcoming resistance conferred by this allele by new bymovirus isolates may be rather likely in contrast to the other loss-of-function alleles (*rym11*-*a*, *b*, *c*, *d*, and *f*), but this is similar to the situation observed for several *HvEIF4E* alleles (Kühne et al. 2003; Stein et al. 2005). Complete knock-out of the *HvPDIL5*-*1* gene likely terminates the co-evolution between bymoviruses and this host factor gene.

Therefore, we postulate that the specific biological functions of both host factors *HvPDIL5*-*1* and *HvEIF4E* has driven the two observed distinct patterns of gene diversity. Importantly, the highest diversity of the resistance alleles for both host factor genes was found in East Asia, a region with high incidence of barley yellow mosaic virus disease (Kühne 2009).

### Did natural selection for bymovirus resistance also occur in European cultivated barley?

The barley yellow mosaic virus disease occurs in a majority of European countries (Kühne 2009). Given the predominance of resistance-conferring alleles in East-Asian accessions, it is unclear whether natural selection for bymovirus resistances also independently happened in Europe, perhaps even before the active breeding for bymovirus resistance in barley had been initiated (since 1980s). No *HvPDIL5*-*1* haplotypes conferring bymovirus resistance (*rym1/11*) were found in the current collection of European barley accessions, supporting the hypothesis that *rym1/11* evolved in East Asia. Three *HvEIF4E* haplotypes (hap-*rym4*, XXII, and XL) that also confer bymovirus resistance were found in 15 of 304 European barley accessions. The eleven historical accessions (landraces) containing hap-*rym4* or hap-XL were particularly collected before 1970 s from the Balkans (not shown), a region where the bymovirus disease was not reported until the early 1990s (Katis et al. 1997). The remaining four accessions carrying hap-XXII were from countries of Western and Northern Europe. The same haplotype (hap-XXII) was found also in Central and East Asia. In some geographic regions, where the barley yellow mosaic virus disease was not reported, twelve accessions (including wild and domesticated) showing bymovirus resistance were found. While there was an enrichment of *HvEIF4E* haplotypes conferring bymovirus resistance in European barley germplasm, from the results of the present study we cannot confidently conclude whether the resistance-conferring mutations in hap-XXII, and hap-XL originated in Europe or were introduced to Europe from elsewhere with the seed trade.

#### Author contribution statement

P.Y., K.K., A.G., F.O. and N.S. designed this research; P.Y., A.H., B.H. and B.K. performed experiments; P.Y. analyzed data; P.Y. and N.S. wrote this manuscript.

## Electronic supplementary material

Below is the link to the electronic supplementary material.

Supplementary material 1 (DOCX 1151 kb)

Supplementary material 2 (XLSX 190 kb)
