# Supplementary material for: Sequence diversification in recessive alleles of two host factor genes suggests adaptive selection for bymovirus resistance in cultivated barley from East Asia
Source: Theor Appl Genet. 2016 Nov 9;130(2):331–44. doi: 10.1007/s00122-016-2814-z (PMC5263206; doi:10.1007/s00122-016-2814-z)
Supplement: Supplementary file 1 — Supplementary material 1 (DOCX 1151 kb) [file 122_2016_2814_MOESM1_ESM.docx]

Supplementary Fig. 1 Collection sites of wild (A) and domesticated (B) barley accessions. Each dot shows a particular collection site.


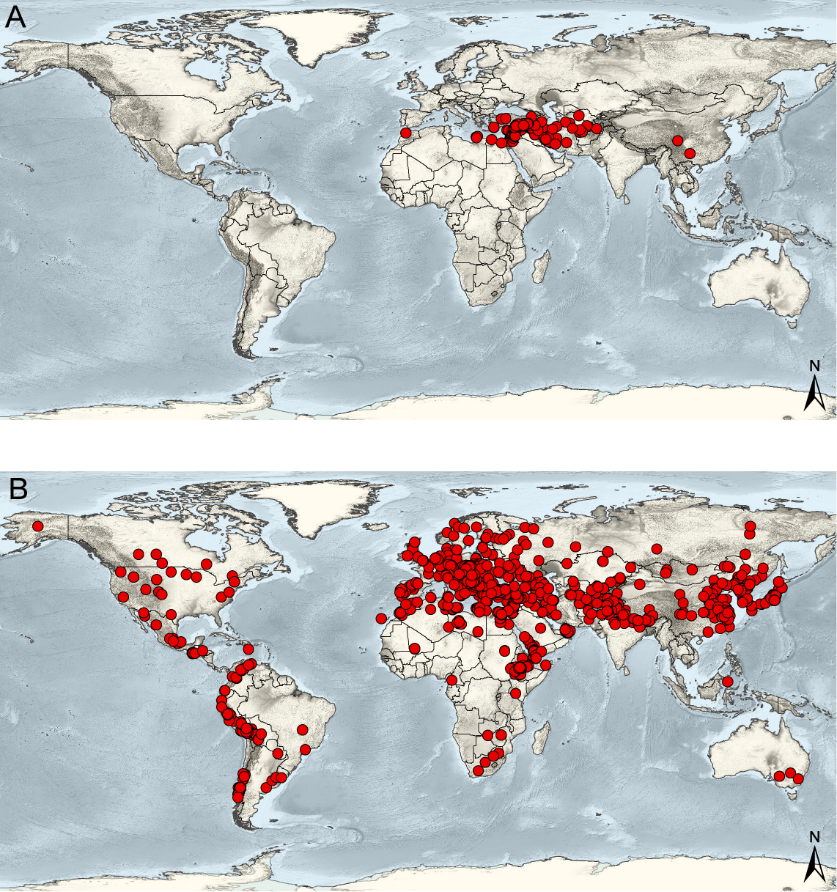


Supplementary Table 1 Summary of the geographic origins of the barley accessions used in this study

| Geographic regions | Countries | Number of countries | *Hs* | *Hv* | *HvPDIL5-1* | *HvEIF4E* | *HvGT43* | *HvMCT-1* |
| --- | --- | --- | --- | --- | --- | --- | --- | --- |
| Africa | Algeria, Cameroon, Egypt, Ethiopia, Kenya, Libya, Mali, Morocco, South Africa, Sudan, Tunisia, Zimbabwe | 12 | 6 | 254 | 246 | 140 | 35 | 32 |
| Americas | Argentina, Bolivia, Brazil, Canada, Chile, Colombia, Ecuador, Guatemala, Honduras, Mexico, Paraguay, Peru, Uruguay, USA, Venezuela | 15 | 0 | 271 | 231 | 204 | 27 | 37 |
| Central Asia | Afghanistan, Bhutan, India, Kazakhstan, Kyrgyzstan, Nepal, Pakistan, Tajikistan, Turkmenistan, Uzbekistan | 10 | 9 | 216 | 186 | 114 | 26 | 38 |
| East Asia | China, Japan, Mongolia, North Korea, South Korea | 5 | 2 | 537 | 444 | 192 | 55 | 70 |
| Europe | Albania, Armenia, Austria, Belarus, Belgium, Bosnia and Herzegovina, Bulgaria, Croatia, Czech, Denmark, Finland, France, Georgia, Germany, Greece, Hungary, Ireland, Italy, Latvia, Lithuania, Macedonia, Moldova, Netherlands, Norway, Poland, Portugal, Romania, Russia, Slovakia, Slovenia, Spain, Sweden, Switzerland, UK, Ukraine | 35 | 2 | 832 | 751 | 306 | 59 | 70 |
| Near East | Azerbaijan, Cyprus, Iran, Iraq, Israel, Jordan, Lebanon, Oman, Saudi Arabia, Syria, Turkey, Yemen | 12 | 340 | 415 | 693 | 434 | 204 | 199 |
| Oceania | Australia | 1 | 0 | 17 | 16 | 14 | 7 | 7 |
| Unknown |  |  | 6 | 15 | 21 | 6 | 0 | 0 |
| Total |  | 89 | 365 | 2557 | 2588 | 1410 | 413 | 453 |

Supplementary Table 2 Geo-referenced wild and domesticated barley accessions (separate excel file)

Supplementary Table 3 The primers used in this study

| Primers | Primer sequence (5’ to 3’) | References |
| --- | --- | --- |
| PDI_45_2138_f | GTATCCGCCTTCTCCTCGTC | Yang et al. 2014b |
| PDI_45_2138_r | AGCATCCAAAGTTGCCTGAC | Yang et al. 2014b |
| EIF4E_m56s | CGCCCGTCCGTCCTAGAAAAG | Yang et al. 2014a |
| EIF4E_309as | GGTAAGAGAGGAACGAATCGGAC | Yang et al. 2014a |
| EIF4E_1659s | CACCGTGGCTTGTCTTCAGCAGG | Yang et al. 2014a |
| EIF4E_2394as | CACAGTGAAGGGCTCACGCTCAG | Yang et al. 2014a |
| EIF4E_4857s | ACCTGGGTAAATGCTATCACG | Stein et al. 2005 |
| EIF4E_5396as | TTATGGAGTACAACGACGACAAACAC | Stein et al. 2005 |
| MCT-1_151s | AATCGCCGCTCACCATGTT | This study |
| MCT-1_978as | TGCATTCTGACTTTTGAAGTGG | This study |
| MCT-1_910s | GCCTGCCCTTAATGAGACTG | This study |
| MCT-1_1719as | CCACACCCATTGCTGAAAAT | This study |
| MCT-1_1617s | TCTCAAGCAAGCGTTCATGT | This study |
| MCT-1_2437as | AGCAACCATGAGTTCCAAGG | This study |
| MCT-1_2334s | TGGCAATTGGATTCACAAAG | This study |
| MCT-1_3146as | CAGTTCCATATGGCCTTGC | This study |
| GT43-cDNA_124s | TCGGTGGTCTGTTTCATGG | This study |
| GT43-cDNA_765as | AAAAAGCGCAGCTCGTACAC | This study |
| GT43-cDNA_607s | CCACCTGACGTACAAGGAGAA | This study |
| GT43-cDNA_1297as | ACACCTTACTGGTGCGATCC | This study |
| C5_11_GT43_1_f | AGACAGCCGTGCTAGCTTCTA | Yang et al. 2014b |
| C5_11_GT43_1_r | GGAGAGTGATTGAGCGAGTGA | Yang et al. 2014b |
| C5_11_GT43_3_f | GACGCGAGAATGTATTTGCTT | Yang et al. 2014b |
| C5_11_GT43_3_r | TAGCGAGAGGGTGTGCTTTAG | Yang et al. 2014b |
| C5_11_GT43_4_f | AGCTCCGTGTTGGTGGTTAG | Yang et al. 2014b |
| C5_11_GT43_4_r | GCTTGTACCCGCCTTCTATTC | Yang et al. 2014b |
| VPg_fwd | TACCGCAATCTGAAGCAAGAAT | This study |
| VPg_rwd | CTGTATGTGCGCAGGAGCTAT | This study |

Supplementary Table 4 The identified naturally occurring *HvPDIL5-1* haplotypes

| Haplotypes | Sequence variations | Number of accessions | Frequency of haplotypes in population | Phenotype to BaMMV-ASL | Description |
| --- | --- | --- | --- | --- | --- |
| I | Wild type | 2446 | 0.94513 | S | Wild type |
| II | 315’-331’, 17-bp deletion | 30 | 0.01159 | R | *rym11-b*, pre-stop |
| III | A240G | 34 | 0.01314 | S | Synonymous |
| IV | A138T | 4 | 0.00155 | S | Synonymous |
| V | 60’-74’, 15-bp deletion | 7 | 0.00270 | S | 5 amino acids deletion, in frame |
| VI | C411T | 1 | 0.00039 | S | Synonymous |
| VII | G182A | 4 | 0.00155 | R | *rym11-d*, pre-stop |
| VIII | 185’, 1-bp deletion | 1 | 0.00039 | R | *rym11-c*, pre-stop |
| IX | T392C | 1 | 0.00039 | R | *rym11-e*, Leu131Pro |
| X | C56A | 3 | 0.00116 | S | Ala19Asp |
| XI | T172C | 9 | 0.00348 | S | Cys58Arg |
| XII | 288 | 3 | 0.00116 | S | Synonymous |
| XIII | A138T; C374G | 9 | 0.00348 | S | Synonymous; Pro125Arg |
| XIV | G51A | 4 | 0.00155 | S | Synonymous |
| XV | G63A | 3 | 0.00116 | S | Synonymous |
| XVI | A66C; T407C | 2 | 0.00077 | S | Synonymous; Leu138Ser |
| XVII | C26A | 3 | 0.00116 | S | Ser9Tyr |
| XVIII | A239G | 1 | 0.00039 | R | Glu80Gly |
| XIX | T213A | 5 | 0.00193 | S | Synonymous |
| XX | G277A | 5 | 0.00193 | S | Gly93Ser |
| XXI | T233C; T407C | 1 | 0.00039 | S | Val78Ser; Leu138Ser |
| XXII | G382C | 1 | 0.00039 | S | Val128Leu |
| XXIII | G349A | 1 | 0.00039 | S | Glu117Lys |
| XXIV | C237T | 2 | 0.00077 | S | Synonymous |
| XXV | G9A | 2 | 0.00077 | S | Synonymous |
| XXVI | T407C | 1 | 0.00039 | Unknown | Leu138Ser |
| XXVII | G70T | 1 | 0.00039 | S | Ala24Ser |
| XXVIII | 299-bp deletion | 1 | 0.00039 | R | *rym11-a*, -567’ to 789’ deletion, no transcript |
| XXIX | G256A | 1 | 0.00039 | R | *rym11-f*, pre-stop |
| XXX | G238T | 2 | 0.00077 | Unknown | Glu80Lys |

Phenotype of each haplotype was given in detail in Supplementary Table 10. S, Susceptibility; R, Resistance.

Supplementary Table 5 The identified naturally occurring *HvEIF4E* haplotypes

| Haplotypes | Number of accessions | Frequency of haplotypes in population | Phenotype to BaMMV-ASL | Description |
| --- | --- | --- | --- | --- |
| wt0A | 805 | 0.57092 | S | Wild type |
| wt0B | 44 | 0.03121 | S | Non-synonymous, susceptible to BaMMV-ASL |
| *rym4* | 8 | 0.00567 | R | *rym4*, resistance to BaMMV, BaYMV-I |
| *rym5* | 1 | 0.00071 | R | *rym5*, resistance to BaMMV, BaYMV-I, BaYMV-II |
| *I* | 248 | 0.17589 | S | Non-synonymous |
| *II* | 26 | 0.01844 | S | Non-synonymous |
| *III* | 36 | 0.02553 | Unknown | Non-synonymous |
| *IV* | 17 | 0.01206 | S | Non-synonymous |
| *V* | 3 | 0.00213 | S | Non-synonymous |
| *VI* | 10 | 0.00709 | S | Non-synonymous |
| *VII* | 9 | 0.00638 | S | Non-synonymous |
| *VIII* | 3 | 0.00213 | S | Non-synonymous |
| *IX* | 1 | 0.00071 | R | Non-synonymous |
| *X* | 1 | 0.00071 | R | Non-synonymous |
| *XI* | 25 | 0.01773 | S | Non-synonymous |
| *XII* | 24 | 0.01702 | Unknown | Non-synonymous |
| *XIII* | 5 | 0.00355 | R | Non-synonymous |
| *XIV* | 17 | 0.01206 | R | Non-synonymous |
| *XV* | 4 | 0.00284 | R | Non-synonymous |
| *XVI* | 6 | 0.00426 | Unknown | Non-synonymous |
| *XVII* | 5 | 0.00355 | R | Non-synonymous |
| *XVIII* | 3 | 0.00213 | S | Non-synonymous |
| *XIX* | 3 | 0.00213 | R | Non-synonymous |
| *XX* | 5 | 0.00355 | S | Non-synonymous |
| *XXI* | 1 | 0.00071 | S | Non-synonymous |
| *XXII* | 10 | 0.00709 | R | Non-synonymous |
| *XXIII* | 2 | 0.00142 | R | Non-synonymous |
| *XXIV* | 1 | 0.00071 | R | Non-synonymous |
| *XXV* | 1 | 0.00071 | R | Non-synonymous |
| *XXVI* | 1 | 0.00071 | S | Non-synonymous |
| *XXVII* | 1 | 0.00071 | R | Non-synonymous |
| *XXVIII* | 5 | 0.00355 | R | Non-synonymous |
| *XXIX* | 2 | 0.00142 | Unknown | Non-synonymous |
| *XXX* | 3 | 0.00213 | Unknown | Non-synonymous |
| *XXXI* | 2 | 0.00142 | R | Non-synonymous |
| *XXXII* | 1 | 0.00071 | S | Non-synonymous |
| *XXXIII* | 1 | 0.00071 | Unknown | Non-synonymous |
| *XXXIV* | 2 | 0.00142 | S | Non-synonymous |
| *XXXV* | 3 | 0.00213 | S | Non-synonymous |
| *XXXVI* | 1 | 0.00071 | S | Non-synonymous |
| *XXXVII* | 1 | 0.00071 | R | Non-synonymous |
| *XXXVIII* | 3 | 0.00213 | S | Non-synonymous |
| *XXXIX* | 2 | 0.00142 | S | Non-synonymous |
| *XL* | 3 | 0.00213 | R | Non-synonymous |
| *XLI* | 1 | 0.00071 | S | Non-synonymous |
| *XLII* | 7 | 0.00496 | S | Non-synonymous |
| *XLIII* | 1 | 0.00071 | Unknown | Non-synonymous |
| *XLIV* | 20 | 0.01418 | S | Synonymous |
| *XLV* | 3 | 0.00213 | R | Non-synonymous |
| *XLVI* | 1 | 0.00071 | S | Synonymous |
| *XLVII* | 2 | 0.00142 | S | Non-synonymous |
| *XLVIII* | 1 | 0.00071 | S | Synonymous |
| *XLIX* | 2 | 0.00142 | S | Non-synonymous |
| *L* | 2 | 0.00142 | S | Non-synonymous |
| *LI* | 1 | 0.00071 | S | Synonymous |
| *LII* | 3 | 0.00213 | S | Synonymous |
| *LIII* | 1 | 0.00071 | S | Synonymous |
| *LIV* | 1 | 0.00071 | S | Synonymous |
| *LV* | 1 | 0.00071 | S | Synonymous |
| *LVI* | 1 | 0.00071 | S | Non-synonymous |
| *LVII* | 2 | 0.00142 | S | Synonymous |
| *LVIII* | 1 | 0.00071 | S | Non-synonymous |
| *LIX* | 1 | 0.00071 | S | Synonymous |
| *LX* | 2 | 0.00142 | S | Non-synonymous |
| *LXI* | 1 | 0.00071 | S | Non-synonymous |

Phenotype of each haplotype was given in detail in Supplementary Table 11. S, Susceptibility; R, Resistance.

Supplementary Table 6 The sequence diversity of *HvEIF4E* haplotypes

wt0A CACCCGCGCCCCCCGTCAACGCCAGACCGGACCGGTGAGGATGCTAAG------------AGTCCAGGCC

wt0B ....................................GAGGATGCTAAG------------.......C..

rym4 .........T.......C..................GAGGATGCTAAG------------...T.G.C..

rym5 ...................G.....G.A........GAGGATGCTAAG------------..........

Hap_I ......T.....................T.......GAGGATGCTAAG------------..........

Hap_II ...................G................GAGGATGCTAAG------------..........

Hap_III ....................................------------------------..........

Hap_VI .T....A.............................GAGGATGCTAAG------------..........

Hap_V ...........................A........GAGGATGCTAAG------------..........

Hap_VI ......T.....................T.......GAGGATGCTAAG------------........G.

Hap_VII .T....A.............A...............GAGGATGCTAAG------------..........

Hap_VIII ............................T.......GAGGATGCTAAG------------..........

Hap_IX .T....A......................A......GAGGATGCTAAG------------..........

Hap_X .T....A.............................GAGGATGCTAAG------------.......A..

Hap_XI ..................G.................GAGGATGCTAAG------------..........

Hap_XII ....................................GAGGATGCTAAG------------......A...

Hap_XIII ...................G................GAGGATGCTAAG------------.......A..

Hap_XIV ...................................AGAGGATGCTAAG------------......A...

Hap_XV ...............A...........A........GAGGATGCTAAG------------......A...

Hap_XVI ...........................A........GAGGATGCTAAG------------.......A..

Hap_XVII ...............A...G.......A........GAGGATGCTAAG------------..........

Hap_XVIII ...................G.......A........GAGGATGCTAAG------------.......A..

Hap_XIX ...................G.......G........GAGGATGCTAAG------------..........

Hap_XX ...................G.......A........GAGGATGCTAAG------------..........

Hap_XXI ...........................G........GAGGATGCTAAG------------......A...

Hap_XXII ....................................GAGGATGCTAAG------------.......A..

Hap_XXIII ...................G....T..A........GAGGATGCTAAG------------..........

Hap_XXIV .............A....................A.GAGGATGCTAAG------------..........

Hap_XXV .........T.......T..................GAGGATGCTAAG------------.....G.C..

Hap_XXVI .................................C..GAGGATGCTAAG------------..........

Hap_XXVII ...................G.......A........GAGGATGCTAAGAGGTCCGACAAAG---......

Hap_XXVIII ....................................GAGGATGCTAAGAGGTCCGACAAAG---......

Hap_XXIX .....................T..............GAGGATGCTAAG------------..........

Hap_XXX ..................................C.GAGGATGCTAAG------------..........

Hap_XXXI ....................................GAGGATGCTAAG------------.....GA...

Hap_XXXII .......................G...........AGAGGATGCTAAG------------......A...

Hap_XXXIII .........T.......C..................GAGGATGCTAAG------------.....GTC..

Hap_XXXIV ....................................GAGGATGCTAAG------------........G.

Hap_XXXV ..........A........G................GAGGATGCTAAG------------..........

Hap_XXXVI ....................T.......C.......GAGGATGCTAAG------------..........

Hap_XXXVII ....................................GAGGATGCTAAGAGGTCCGACAAA......A...

Hap_XXXVIII ..........................AG........GAGGATGCTAAG------------..........

Hap_XXXIX ..........................AG........GAGGATGCTAAG------------........G.

Hap_XL .........T.......C..................GAGGATGCTAAG------------.....G.C..

Hap_XLI ...............................T....GAGGATGCTAAG------------..........

Hap_XLII ..................................T.GAGGATGCTAAG------------..........

Hap_XLIII .................C..................GAGGATGCTAAG------------.....G.C..

Hap_XLIV ..............C.....................GAGGATGCTAAG------------.......C..

Hap_XLV ............T.......................GAGGATGCTAAG------------..........

Hap_XLVI ........A...........................GAGGATGCTAAG------------..........

Hap_XLVII ..............C.....................GAGGATGCTAAG------------..........

Hap_XLVIII ........A..T........................GAGGATGCTAAG------------..........

Hap_XLIX ...........................A......A.GAGGATGCTAAG------------..........

Hap_L ....................T...............GAGGATGCTAAG------------..........

Hap_LI ..T..T................T.............GAGGATGCTAAG------------..........

Hap_LII ................................T...GAGGATGCTAAG------------..........

Hap_LIII .......A............................GAGGATGCTAAG------------..........

Hap_LIV ....G...............................GAGGATGCTAAG------------..........

Hap_LV ...T................................GAGGATGCTAAG------------..........

Hap_LVI T...................................GAGGATGCTAAG------------..........

Hap_LVII ................T...................GAGGATGCTAAG------------..........

Hap_LVIII ......................T.......G.....GAGGATGCTAAG------------..........

Hap_LIX ....................................GAGGATGCTAAG------------....T.....

Hap_LX ....................................GAGGATGCTAAG------------.........T

Hap_LXI ..T.................................GAGGATGCTAAG------------..........

Nucleotide positions are given as below.

[1]5 [2]22 [3]39 [4]75 [5]87

[6]105 [7]157 [8]162 [9]165 [10]170

[11]182 [12]192 [13]241 [14]267 [15]277

[16]278 [17]285 [18]353 [19]358 [20]359

[21]365 [22]383 [23]432 [24]476 [25]477

[26]478 [27]480 [28]481 [29]483 [30]488

[31]518 [32]524 [33]540 [34]541 [35]584

[36]593 [37]598 [38]599 [39]600 [40]601

[41]602 [42]603 [43]604 [44]605 [45]606

[46]607 [47]608 [48]609 [49]610 [50]611

[51]612 [52]613 [53]614 [54]615 [55]616

[56]617 [57]618 [58]619 [59]620 [60]621

[61]622 [62]624 [63]625 [64]626 [65]627

[66]629 [67]634 [68]635 [69]638 [70]651

Supplementary Table 7 The identified naturally occurring *HvGT43* haplotypes

| Haplotypes | Mutation loci on CDS | Number of accessions | Frequency of haplotypes in total accessions | Description |
| --- | --- | --- | --- | --- |
| I | Wild type | 231 | 0.55932 | Wild type |
| II | G294A; G798T | 48 | 0.11622 | Synonymous; Synonymous |
| III | G294A | 107 | 0.25908 | Synonymous |
| IV | C777T | 2 | 0.00484 | Synonymous |
| V | G294A; G804T | 1 | 0.00242 | Synonymous; Synonymous |
| VI | C283T; G294A; G798T | 1 | 0.00242 | Synonymous; Synonymous; Synonymous |
| VII | G798T | 8 | 0.01937 | Synonymous |
| VIII | G294A; G321A | 1 | 0.00242 | Synonymous; Synonymous |
| IX | G178A; G294A | 1 | 0.00242 | Val60Met; Synonymous |
| X | G381T | 2 | 0.00484 | Synonymous |
| XI | G681A | 2 | 0.00484 | Synonymous |
| XII | C60T; T318C | 3 | 0.00726 | Synonymous; Synonymous |
| XIII | C786T | 1 | 0.00242 | Synonymous |
| XIV | C579A | 1 | 0.00242 | Synonymous |
| XV | C273T; G294A | 1 | 0.00242 | Synonymous; Synonymous |
| XVI | G1000A | 2 | 0.00484 | Ser334Asn |
| XVII | 1062, 3-bp TGG insertion | 1 | 0.00242 | 355Trp, 1 amino acid insertion |

Supplementary Table 8 The identified naturally occurring *HvMCT-1* haplotypes.

| Haplotypes | Mutation loci on CDS | Number of accessions | Frequency of haplotypes in total accessions | Description |
| --- | --- | --- | --- | --- |
| I | Wild type | 418 | 0.92274 | Wild type |
| II | A380G | 25 | 0.05519 | Glu127Gly |
| III | C468T | 1 | 0.00221 | Synonymous |
| IV | A457G | 4 | 0.00883 | Synonymous |
| V | C471T | 1 | 0.00221 | Lys153Glu |
| VI | G85A | 2 | 0.00442 | Ala29Thr |
| VII | G288A | 1 | 0.00221 | Synonymous |
| VIII | G379A | 1 | 0.00221 | Glu127Lys |

Supplementary Table 9 Statistics of sequence diversity of *HvPDIL5-1*, *HvGT43*, *HvEIF4E* and *HvMCT-1* in a subset of barley accessions

| Genes | No. of  fl-ORFs | Polymorphisms | | | No. of haplotypes | *H.* | *π* | *D*^*^ | *F*^*^ | *Tajima’s D* |
| --- | --- | --- | --- | --- | --- | --- | --- | --- | --- | --- |
|  |  | *LoF* | *Ns* | *S* |  |  |  |  |  |  |
| *PDIL5-1_Hs* | 192 | 0 | 8 | 7 | 15 | 0.22500 | 0.00064 | -2.67986^*^ | -3.02792^**^ | -2.26936^**^ |
| *PDIL5-1_Hv* | 221 | 1 | 1 | 2 | 5 | 0.03600 | 0.00008 | -2.26963 | -2.37727^*^ | -1.46571 |
| *GT43_Hs* | 192 | 0 | 1 | 12 | 14 | 0.56200 | 0.00069 | -1.82707 | -2.11640 | -1.66355 |
| *GT43_Hv* | 221 | 0 | 2 | 3 | 7 | 0.63200 | 0.00080 | -1.73015 | -1.18574 | 0.46450 |
| *eIF4E_Hs* | 182 | 0 | 12 | 9 | 21 | 0.59700 | 0.00137 | -3.08815^*^ | -3.22068^**^ | -2.05605^*^ |
| *eIF4E_Hv* | 270 | 0 | 27 | 0 | 35 | 0.71400 | 0.00262 | -2.48456^*^ | -2.60587^*^ | -1.70547 |
| *MCT-1_Hs* | 182 | 0 | 3 | 3 | 7 | 0.21300 | 0.00040 | -3.03527^*^ | -3.03194^**^ | -1.61976 |
| *MCT-1_Hv* | 270 | 0 | 3 | 0 | 4 | 0.09900 | 0.00018 | -2.33181^*^ | -2.33493^*^ | -1.21938 |

Supplementary Table 10 Accessions carrying *HvPDIL5-1* haplotypes inoculated by BaMMV-ASL

| *PDIL5-1* haplotypes | Accession name | Taxonomy | FAO country code | BaMMV-ASL | Phenotype of *PDIL5-1* haplotype | *EIF4E* haplotypes | Comment |
| --- | --- | --- | --- | --- | --- | --- | --- |
| *I* |  |  |  |  | S |  | Yang et al. 2014b |
| *II (rym11-b)* |  |  |  |  | R |  | Yang et al. 2014b |
| *III* | HOR2932 | *Hv.* | ETH | S | S | n.d. | Synonymous |
|  | HOR6360 | *Hv.* | ETH | S |  | n.d. | Synonymous |
|  | HOR10364 | *Hv.* | ITA | S |  | n.d. | Synonymous |
|  | BCC1367 | *Hv.* | DEU | S |  | n.d. | Synonymous |
|  | BCC1411 | *Hv.* | DEU | S |  | n.d. | Synonymous |
| *IV* |  |  |  |  | S | n.d. | Synonymous; Not tested |
| *V* | HOR9614 | *Hv.* | GEO | S | S | n.d. |  |
|  | HOR9615 | *Hv.* | GEO | S |  | n.d. |  |
|  | HOR10360 | *Hv.* | GEO | S |  | n.d. |  |
|  | HOR10754 | *Hv.* | GEO | S |  | n.d. |  |
| *VI* |  |  |  |  | S | n.d. | Synonymous; Not tested |
| *VII (rym11-d)* |  |  |  | R | R |  | Yang et al. 2014b |
| *VIII (rym11-c)* |  |  |  | R | R |  | Yang et al. 2014b |
| *IX (rym11-e)* |  |  |  | R | R |  | Yang et al. 2014a |
| *X* | HOR7583 | *Hv.* | PAK | R? | S | n.d. | Confirmed by allelism test; Not shown |
| *XI* | HOR1107 | *Hv.* | TUR | S | S | n.d. |  |
|  | HOR1099 | *Hv.* | TUR | S |  | n.d. |  |
|  | HOR1097 | *Hv.* | TUR | S |  | n.d. |  |
|  | HOR1070 | *Hv.* | TUR | S |  | n.d. |  |
|  | HOR1055 | *Hv.* | TUR | S |  | n.d. |  |
|  | HOR1048 | *Hv.* | TUR | S |  | n.d. |  |
|  | HOR1047 | *Hv.* | TUR | S |  | n.d. |  |
|  | HOR1045 | *Hv.* | TUR | S |  | n.d. |  |
| *XII* |  |  |  |  | S |  | Synonymous; Not tested |
| *XIII* | FT001 | *Hs.* | ISR | S | S | *wt0A* |  |
|  | FT580 | *Hs.* | TUR | S |  | *wt0A* |  |
| *XIV* |  |  |  |  | S |  | Synonymous; Not tested |
| *XV* |  |  |  |  | S |  | Synonymous; Not tested |
| *XVI* | FT187 | *Hs.* | ISR | S | S | *wt0A* |  |
| *XVII* | FT099 | *Hs.* | ISR | S | S | *LXI* |  |
| *XVIII* | FT027 | *Hs.* | ISR | R | R | *XLIV* | *EIF4E* hap-XLIV is susceptible |
| *XIX* |  |  |  |  | S |  | Synonymous; Not tested |
| *XX* | FT255 | *Hs.* | TUR | S | S | *wt0B* |  |
|  | FT667 | *Hs.* | TUR | S |  | *wt0B* |  |
| *XXI* | FT165 | *Hs.* | ISR | S | S | *wt0A* |  |
| *XXII* | FT197 | *Hs.* | ISR | S | S | *LII* | *EIF4E* hap-LII is susceptible |
| *XXIII* | FT626 | *Hs.* | JOR | S | S | *wt0A* | *EIF4E* hap-wt0A is susceptible |
| *XXIV* |  |  |  |  | S |  | Synonymous; Not tested |
| *XXV* |  |  |  |  | S |  | Synonymous; Not tested |
| *XXVI* | FT173 | *Hs.* | ISR | Unknown | Unknown | *XI* | Seed not available, Not tested |
| *XXVII* | FT167 | *Hs.* | ISR | S | S | *wt0B* | *EIF4E* hap-wt0B is susceptible |
| *XXVIII (rym11-a)* | HOR1363 | *Hv.* | TUR | R | R | *wt0A* | Yang et al. 2014, PNAS |
| *XXIX (rym11-f)* | CIho14399 | *Hv.* | JPN | R | R | *XVI* | *rym11-f* is resistant |
| *XXX* |  |  |  |  | Unknown | n.d. | Not tested |

*Hs.* = *Hordeum spontaneum*, *Hv.* = *Hordeum vulgare*. R, Resistance, S, Susceptibility. n.d. – not determined.

Supplementary Table 11 Accessions carrying *HvEIF4E* haplotypes inoculated by BaMMV-ASL

| *EIF4E* haplotypes | Tested accession | Taxonomy | FAO country code | BaMMV-ASL | Phenotype of *EIF4E* haplotype | *PDIL5-1* haplotypes | Comment |
| --- | --- | --- | --- | --- | --- | --- | --- |
| *wt0A* | Morex | *Hv.* | USA | S | S | *I* | Stein et al. 2005; Hofinger et al. 2011 |
| *wt0B* | Alraune | *Hv.* | GER | S | S | *I* | Stein et al. 2005; Hofinger et al. 2011 |
| *rym4* | Ragusa | *Hv.* | HRV | R | R | *I* | Stein et al. 2005 |
| *rym5* | Moku 49 | *Hv.* | CHN | R | R | *VIII, rym11-d* | Stein et al. 2005 |
| *I* | CIho3100 | *Hv.* | ARG | S | S | n.d. |  |
|  | CIho6199 | *Hv.* | CHL | S |  | *I* |  |
|  | PI246785 | *Hv.* | COL | S |  | *I* |  |
| *II* | CIho7556 | *Hv.* | ARG | S | S | n.d. |  |
| *III* | PI270599 | *Hv.* | PER | R | Unclear | n.d. | *PDIL5-1* haplotype is not determined |
|  | PI477845 | *Hv.* | BOL | R |  | n.d. | *PDIL5-1* haplotype is not determined |
| *IV* | HOR6145 | *Hv.* | ETH | S | S | *I* |  |
|  | PI478428 | *Hv.* | BOL | S |  | n.d. |  |
|  | PI477769 | *Hv.* | PER | S |  | *I* |  |
| *V* | HOR3338 | *Hv.* | UKR | S | S | *I* |  |
|  | PI467366 | *Hv.* | MEX | S |  | n.d. |  |
| *VI* | BCC1355 | *Hv.* | FRA | S | S | *I* |  |
|  | PI55527 | *Hv.* | TUN | S |  | *I* |  |
| *VII* | PI606296 | *Hv.* | YEM | S | S | *I* |  |
|  | HOR7935 | *Hv.* | ETH | S |  | *I* |  |
| *VIII* | CIho3694 | *Hv.* | EGY | S | S | *I* |  |
| *IX* | CIho5021 | *Hv.* | ETH | R | R | *I* | *PDIL5-1* hap-I is susceptible |
| *X* | CIho4359 | *Hv.* | ETH | R | R | *III* | *PDIL5-1* hap-III is susceptible |
| *XI* | PI57634 | *Hv.* | EGY | S | S | *I* |  |
| *XII* |  |  |  |  | Unclear |  | Not tested |
| *XIII* | PI39507 | *Hv.* | CHN | R | R | n.d. |  |
|  | CIho2472 | *Hv.* | CHN | R |  | *I* | *PDIL5-1* hap-I is susceptible |
| *XIV* | PI26459 | *Hv.* | JPN | R | R | *I* | *PDIL5-1* hap-I is susceptible |
|  | PI39498 | *Hv.* | CHN | R |  | n.d. |  |
| *XV* | HOR11707 | *Hv.* | JPN | R | R | *I* | *PDIL5-1* hap-I is susceptible |
|  | PI31901 | *Hv.* | JPN | R |  | *I* | *PDIL5-1* hap-I is susceptible |
|  | PI31902 | *Hv.* | JPN | R |  | n.d. | *PDIL5-1* haplotype is not determined |
| *XVI* | CIho14399 | *Hv.* | JPN | R | Unclear | *XXIX, rym11-f* | *rym11-f* is resistant |
|  | PI26457 | *Hv.* | JPN | R |  | n.d. |  |
| *XVII* | BCC477 | *Hv.* | CHN | R | R | *I* | *PDIL5-1* hap-I is susceptible |
|  | PI31105 | *Hv.* | CHN | R |  | n.d. |  |
|  | PI31106 | *Hv.* | CHN | R |  | n.d. |  |
| *XVIII* | PI39521 | *Hv.* | CHN | S | S | *I* |  |
| *XIX* | BCC484 | *Hv.* | CHN | R | R | *II, rym11-b* | *rym11-b* is resistant |
|  | CIho2461 | *Hv.* | CHN | R |  | *I* | *PDIL5-1* hap-I is susceptible |
|  | PI80813 | *Hv.* | JPN | R |  | *I* | *PDIL5-1* hap-I is susceptible |
| *XX* | PI31384 | *Hv.* | CHN | S | S | n.d. |  |
|  | PI87752 | *Hv.* | KOR | S |  | n.d. |  |
| *XXI* | PI57023 | *Hv.* | JPN | S | S | *I* |  |
| *XXII* | CIho2320 | *Hv.* | CHN | R | R | *I* | *PDIL5-1* hap-I is susceptible |
| *XXIII* | PI34129 | *Hv.* | CHN | R | R | n.d. |  |
|  | PI72012 | *Hv.* | CHN | R |  | *I* | *PDIL5-1* hap-I is susceptible |
| *XXIV* | HOR11594 | *Hv.* | KOR | R | R | *II, rym11-b* | Confirmed by Allelism test; Not shown |
|  | PI87766 | *Hv.* | KOR | R |  | n.d. |  |
| *XXV* | PI155097 | *Hv.* | JPN | R | R | n.d. | R-allele, Dragan et al., 2014 |
| *XXVI* | PI61275 | *Hv.* | CHN | S | S | *I* |  |
| *XXVII* | PI87186 | *Hv.* | KOR | R | R | *I* | *PDIL5-1* hap-I is susceptible |
| *XXVIII* | PI315859 | *Hv.* | GBR | R | R | *I* | *PDIL5-1* hap-I is susceptible |
|  | CIho4125 | *Hv.* | AFG | R |  | n.d. |  |
| *XXIX* | PI39365 | *Hv.* | IND | R | Unclear | n.d. | *PDIL5-1* haplotype is not determined |
| *XXX* | CIho14248 | *Hv.* | AFG | R | Unclear | n.d. | *PDIL5-1* haplotype is not determined |
| *XXXI* | PI34127 | *Hv.* | CHN | R | R | n.d. |  |
|  | PI39514 | *Hv.* | CHN | R |  | *I* | *PDIL5-1* hap-I is susceptible |
|  | HOR11076 | *Hv.* | PRK | R |  | *I* | *PDIL5-1* hap-I is susceptible |
| *XXXII* | CIho2459 | *Hv.* | CHN | S | S | *I* |  |
| *XXXIII* | PI94886 | *Hv.* | TUR | R | Unclear | n.d. | *PDIL5-1* haplotype is not determined |
| *XXXIV* | PI24497 | *Hv.* | TKM | S | S | *I* |  |
| *XXXV* | CIho2353 | *Hv.* | TKM | S | S | *I* |  |
| *XXXVI* | PI64022 | *Hv.* | UZB | S | S | n.d. |  |
|  | HOR8659 | *Hv.* | EGY | S |  | *I* |  |
| *XXXVII* | HOR11537 | *Hv.* | CHN | R | R | *I* | *PDIL5-1* hap-I is susceptible |
|  | PI39500 | *Hv.* | CHN | R |  | *I* | *PDIL5-1* hap-I is susceptible |
| *XXXVIII* | PI328678 | *Hv.* | HUN | S | S | n.d. |  |
|  | PI344870 | *Hv.* | MKD | S |  | n.d. |  |
|  | HOR4727 | *Hv.* | TUR | S |  | *I* |  |
| *XXXIX* | PI328598 | *Hv.* | ALB | S | S | *I* |  |
|  | PI344890 | *Hv.* | MKD | S |  | n.d. |  |
| *XL* | HOR1046 | *Hv.* | TUR | R | R | n.d. | Confirmed by Allelism test; not shown |
|  | PI344914 | *Hv.* | BIH | R |  | n.d. |  |
|  | PI264920 | *Hv.* | BIH | R |  | n.d. |  |
| *XLI* | PI265463 | *Hv.* | FIN | S | S | n.d. |  |
| *XLII* | PI58063 | *Hv.* | ESP | S | S | n.d. |  |
| *XLIII* | PI264908 | *Hv.* | GRC | R | Unclear | n.d. | *PDIL5-1* haplotype is not determined |
| *XLIV* |  |  |  |  | S |  | Synonymous; Not tested |
| *XLV* | FT579 | *Hs.* | TUR | R | R | *I* | *PDIL5-1* hap-I is susceptible |
|  | FT751 | *Hs.* | TUR | R |  | *I* | *PDIL5-1* hap-I is susceptible |
| *XLVI* |  |  |  |  | S |  | Synonymous; Not tested |
| *XLVII* | FT614 | *Hs.* | IRN | S | S | n.d. |  |
| *XLVIII* |  |  |  |  | S |  | Synonymous; Not tested |
| *XLIX* | FT387 | *Hs.* | ISR | S | S | *I* |  |
| *L* | FT568 | *Hs.* | AFG | S | S | *I* |  |
|  | FT567 | *Hs.* | TKM | S | S | *I* |  |
| *LI* |  |  |  |  | S |  | Synonymous; Not tested |
| *LII* | FT197 | *Hs.* | ISR | S | S |  | Synonymous |
| *LIII* |  |  |  |  | S |  | Synonymous; Not tested |
| *LIV* |  |  |  |  | S |  | Synonymous; Not tested |
| *LV* |  |  |  |  | S |  | Synonymous; Not tested |
| *LVI* | FT462 | *Hs.* | TUR | S | S | *I* |  |
| *LVII* |  |  |  |  | S |  | Synonymous; Not tested |
| *LVIII* | FT284 | *Hs.* | IRN | S | S | *I* |  |
| *LIX* |  |  |  |  | S |  | Synonymous; Not tested |
| *LX* | FT113 | *Hs.* | ISR | S | S | *I* |  |
|  | FT123 | *Hs.* | ISR | S |  | *I* |  |
| *LXI* | FT099 | *Hs.* | ISR | S | S | *XVII* |  |

*Hs.* = *Hordeum spontaneum*, *Hv.* = *Hordeum vulgare*. R, Resistance, S, Susceptibility. n.d. – not determined.
